# Supplementary material for: Homophilic Interaction of CD147 Promotes IL-6-Mediated Cholangiocarcinoma Invasion via the NF-κB-Dependent Pathway
Source: Int J Mol Sci. 2021 Dec 16;22(24):13496. doi: 10.3390/ijms222413496 (PMC8706168; doi:10.3390/ijms222413496)
Supplement: Supplementary file 1 [file ijms-22-13496-s001.zip › ijms-1480580-supplementary.pdf]

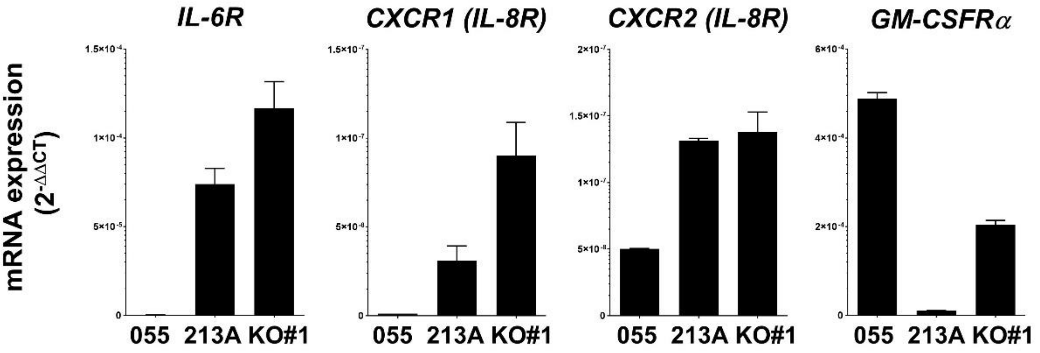

Supplementary Figure S1 IL-6, IL-8 and GM-CSF receptor expressions in CCA cells. Expressions of IL-6 receptor (*IL-6R*), IL-8 receptor (*CXCR1* and *CXCR2*), and GM-CSF receptor (*GM-CSFRα*) were measured by real-time RT-PCR. The expressions are expressed as mean ± SEM of $2^{-\Delta\Delta CT}$ from representative experiments. 055 = KKU-055, 213A = KKU-213A, KO#1 = CD147 KO#1.

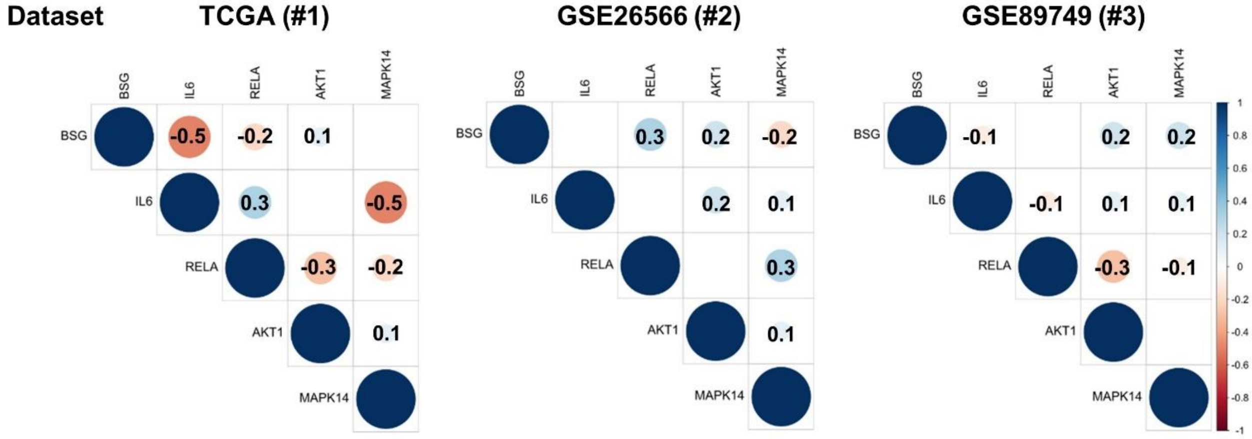

Supplementary Figure S2 The correlations between CD147 (BSG), IL-6, p65 (RELA), Akt (AKT1), and p38 (MAPK14) in CCA clinical samples obtained from 3 different datasets. Numbers represent the correlation scores. Negative correlations are presented in orange circles and numbers < 0. Only correlation scores with p<0.05 are presented.
